# A High-Resolution MRI Study of Relationship between Remodeling Patterns and Ischemic Stroke in Patients with Atherosclerotic Middle Cerebral Artery Stenosis

**DOI:** 10.3389/fnagi.2017.00140

**Published:** 2017-05-09

**Authors:** Dan-Feng Zhang, Yu-Chen Chen, Huiyou Chen, Wei-Dong Zhang, Jun Sun, Cun-Nan Mao, Wen Su, Peng Wang, Xindao Yin

**Affiliations:** ^1^Department of Radiology, Nanjing First Hospital, Nanjing Medical UniversityNanjing, China; ^2^School of Medicine, Nanjing UniversityNanjing, China

**Keywords:** high-resolution MRI, remodeling pattern, ischemic stroke, middle cerebral artery stenosis, atherosclerosis

## Abstract

**Purpose:** Recently, high-resolution magnetic resonance imaging (HR-MRI) has been used to depict the wall characteristics of the intracranial arteries. The aim of this study was to explain the relationship between the remodeling patterns and acute ischemic stroke in patients with atherosclerotic middle cerebral artery (MCA) stenosis using HR-MRI.

**Materials and Methods:** From August 2015 to May 2016, we prospectively screened 33 consecutive patients with unilateral MCA stenosis using time-to-flight MR angiography, including 15 patients with symptomatic MCA stenosis and 18 patients with asymptomatic MCA stenosis. Among them, 14 patients were diagnosed as positive remodeling (PR) and 19 as negative remodeling or non-remodeling. The cross-sectional images of the stenotic MCA wall on HR-MRI including T1WI, T2WI, and PDWI were compared between the symptomatic group and the asymptomatic group as well as the PR group and the non-PR group, based on the vessel area, lumen area, wall area, plaque area, degree of stenosis, remodeling index, and NIHSS score.

**Results:** The symptomatic group had larger wall area (*P* = 0.040), plaque area (*P*<0.001), degree of stenosis (*P* = 0.038), remodeling index (*P* < 0.001), and NIHSS score (*P* = 0.003) as well as smaller lumen area (*P* = 0.001) than the asymptomatic group. In addition, more PR patients were observed in symptomatic group. The PR group had larger plaque area (*P* = 0.014) and NIHSS score (*P* = 0.037) than the non-PR group. Demographic and clinical characteristics between the symptomatic group and the asymptomatic group, the PR group and the non-PR group showed no statistical difference.

**Conclusion:** The current study suggests that the HR-MRI has emerged as a promising tool to detect the characteristics of intracranial arteries wall and reveal the relationship between remodeling patterns and ischemic stroke. The PR is an unsafe remodeling way and is prone to cause acute ischemic stroke.

## Introduction

Intracranial atherosclerotic disease was one of the major causes of ischemic stroke throughout the world (Amarenco et al., 1994; Arenillas, 2011; Banerjee and Chimowitz, 2017), accounting for about 10% of transient ischemic attack and 30%-50% of ischemic stroke (Qureshi et al., 2009), and it was the most common factor in the Asian population (Arenillas, 2011; Yang et al., 2016). Moreover, MCA was the commonest atherosclerotic stenotic location in Asian (Wong et al., 2002). For many years, it was thought that stenotic grade of MCA was the most accurate reflection of the ischemic stroke risk (Lehrke et al., 2009). However, prior studies have suggested that degree of stenosis had no statistical significance between symptomatic and asymptomatic groups in moderate to severe MCA stenosis (Xu et al., 2010; Shi et al., 2012). Thus, it was thought that intracranial atherosclerosis should be evaluated with not only stenotic grade but also characteristics of vessel wall (Chung et al., 2012; Zhu et al., 2013; Zhao et al., 2015).

Arterial remodeling patterns including positive remodeling (PR) and negative remodeling (NR) were raised in earlier studies regarding the coronary arteries (Glagov et al., 1987). The PR caused the vessel enlargement and alleviated vessel stenosis to some extent, while the NR resulted in constriction of the lumen. However, PR was thought to be an unsafe way for vessel stenosis. Shi et al. (2012) reported that microembolic signals were observed more frequently in the PR group than non-PR group at the stenotic MCA, which demonstrated more vulnerable plaques (Shi et al., 2012). Schoenhagen et al. detected that the PR was obviously associated with acute cardiac symptoms while the NR was more common in patients with stable angina (Schoenhagen et al., 2000). Similarly, PR in carotid arteries was more common in patients with cerebral ischemic symptoms (Hardie et al., 2007). Moreover, since the MCA had similar structures and components with the coronary arteries and carotid arteries, arterial remodeling of MCA might share the same vascular biological features (Shi et al., 2012). Nevertheless, the direct relationships between the MCA and the arterial remodeling still remain unclear.

Currently, common techniques used for cerebrovascular assessment mainly contain the digital subtraction angiography (DSA), computed tomography angiography (CTA), and conventional magnetic resonance angiography (MRA); however, these methods cannot reveal the characteristics of vessel wall clearly and accurately while the DSA and CTA accompany with radiation damage (Xu et al., 2015). Fortunately, HR-MRI was thought to be a potential tool to characterize the vessel wall of MCA and evaluate the degree of MCA stenosis simultaneously (Ryu et al., 2009). The HR-MRI could depict margin and atherosclerotic plaques in stem of MCA, *in vivo*, without any injuries (Xu et al., 2010; Mossa-Basha et al., 2016; Zhu et al., 2016), which has been used to study vessel wall of MCA through many researches. Meanwhile, the HR-MRI could offer more advantages such as higher signal-to-noise ratio and minimal scan duration compared with conventional MRI (Chung et al., 2012). Nonetheless, few studies had reported the direct relationships between acute stroke in the distribution of stenotic MCA and the vessel wall characteristics, as well as the correlations between remodeling patterns and neurological deficits in acute ischemic stroke patients. Therefore, the current study aims to comprehensively assess the specific vessel wall characteristics and the remodeling patterns in ischemic stroke patients with atherosclerotic MCA stenosis by using 3.0-Tesla HR-MRI.

## Materials and Methods

### Subjects

Between August 2015 and May 2016, 33 consecutive patients with suspected MCA atherosclerotic diseases from Department of Neurology (Nanjing First Hospital, Nanjing Medical University) were recruited. Demographic and clinical characteristics of patients were obtained from their medical record before the MR scan, including sex, age, smoking, alcoholism, hypertension, diabetes, blood glucose, HbA1c, total cholesterol, low-density lipoprotein (LDL), high-density lipoportein (HDL), triglycerides, homocysteine, phospholipase-A2, white blood cell count, time from admission to HR-MRI examination, and the National Institutes of Health Stroke Scale (NIHSS). All patients underwent standard MR protocols including axial plain scan T1WI, T2WI, T2-fluid attenuated inversion recovery (FLAIR), diffusion-weighted imaging (DWI), time-of-flight MRA (TOF-MRA) in 1 week after admission. The patients had clinical symptoms, such as dizziness, alalia, limb weakness, or sleepy. Symptomatic patients were considered for inclusion if there was hyperintense signal on DWI in the distribution of the stenotic MCA. Asymptomatic patients were considered for inclusion if there was no hyperintense signal on DWI in the distribution of the stenotic MCA. The criteria of patients enrollment in this study included: (1) single MCA M1 segment stenosis >30% showed on MRA; (2) two or more athersclerotic risk factors; (3) without contraindications to MR scan; (4) the stenosis of ipsilateral internal carotid artery less than 50%; (5) the quality of pictures could be used for diagnosis and analysis; (6) without non-atherosclerotic vasculopathy, such as vasculitis, moyamoya disease, dissection, cerebral hemorrhage, tumor etc.; (7) no evidence of arterial fibrillation, cardioembolism. Approval for the study was obtained by the Ethics Committee of the Nanjing Medical University. Written informed consent from all the patients was obtained.

### HR-MRI Protocol

All patients underwent the HR-MRI of stenotic MCA using a 3-Tesla MR scanner (Ingenia, Philips Medical Systems, Netherlands) with an 8-channel receiver array head coil. TOF-MRA was reconstructed to determine the blood vessel architecture, which was then used for positioning to ensure the stenosis of MCA. We performed HR-MRI scanning, including black-blood T1WI, T2WI and PDWI, perpendicular to M1 segment of MCA. The imaging sequences of HR-MRI were applied with following parameters: (1) TOF-MRA: repetition time (TR), 22 ms; echo time (TE), 3.45 ms; number of excitation (NEX), 1; field of view (FOV), 200 mm × 84 mm; matrix size, 332 × 227; slice thickness, 0.6 mm; slice number, 140; (2) T1WI: TR, 1,000 ms; TE, 9 ms; and NEX, 2; FOV, 80 mm × 80 mm; matrix size, 180 mm × 144 mm; slice thickness, 2.0 mm; slice gap, 0 mm; and slice number, 6; (3) T2WI: TR, 3,000 ms; TE, 80 ms; and NEX, 1; FOV, 80 mm × 80 mm; matrix size, 180 mm × 144 mm; slice thickness, 2.0 mm; slice gap, 0 mm; and slice number, 6; (4) PDWI: TR, 2,000 ms; TE, 9 ms; and NEX, 2; FOV, 80mm × 80 mm; matrix size, 180 mm × 144 mm; slice thickness, 2.0 mm; slice gap, 0 mm; and slice number, 6.

### MR Image Analysis

All parameters measurements were performed on Philips Intellispace Portal workstation. We magnified the short axial PDWI images to 300% and measured the vessel area (VA), lumen area (LA) of MCA at the most narrowed lumen (MNL) and at the reference site. The reference site was the nearest plaque-free or minimally diseased segments proximal to the stenotic MCA. If a proximal reference site was not available, then the neighboring distal site was used instead.

The degree of stenotic MCA on HR-MRI was calculated using the following formula: degree of stenosis = (1- luminal area at the MNL site/ reference lumen area) x 100%. The wall area (WA) = VA–LA, the plaque area (PA) = WA_MNL_–WA_reference_, the remodeling index (RI) = VA_MNL_/VA_reference_. We defined RI ≥ 1.05 as PR, RI ≤ 0.95 as NR, 0.95 < RI < 1.05 as non-remodeling. The measurement work was done by two professional radiologists (W-D Z and JS) who were blinded to clinical details in 2 days after scanning, and then the average value was calculated and applied. The slice for measurement was agreed between two observers.

### Statistical Analysis

All data were analyzed by using SPSS21.0 package (Chicago, IL, USA). Quantitative data was expressed as mean ± standard deviation. Categorical values were summarized using counts and percentages. The *t*-test was used for quantitative data between groups; Fisher exact test was used for categorical variables. Values of *P* < 0.05 were defined as statistical significance.

## Results

### Demographic and Clinical Characteristics

A total of 33 consecutive patients with MCA M1 segment stenosis underwent 3.0 Tesla HR-MRI. Fifteen patients had hyperintense signal on DWI in the distribution of the stenotic MCA, who were divided into the symptomatic group. The asymptomatic group was composed of the others. The differences of sex, age, smoker, alcoholism, hypertension, diabetes, blood glucose, HbA1c, total cholesterol, LDL, HDL, triglycerides, homocysteine, phospholipase-A2, white blood cell count, time from admission to HR-MRI examination, location of stenosis between the symptomatic group and the asymptomatic group showed no statistical significances (**Table 1**).

**Table 1:** Demographic and clinical characteristics of symptomatic group and asymptomatic group.

	Symptomatic group (*n* = 15)	Asymptomatic group (*n* = 18)	*t*-value	*P*-value
Male (%)	12 (80%)	15 (83%)		0.233^a^
Age (years)	67 ± 14	69 ± 10	0.495	0.624
Smoker (%)	8 (53%) 90	11 (61%)		0.733^a^
Alcoholism (%)	7 (47%)	9 (50%)		1.000^a^
Hypertension (%)	12 (80%)	15 (83%)		1.000^a^
Diabetes (%)	9 (60%)	8 (53%)		0.491^a^
Blood glucose (mmol/L)	5.6 ± 1.4	6.3 ± 1.7	1.141	0.263
HbA1c (%)	6.6 ± 1.4	6.5 ± 1.3	–0.018	0.986
Total cholesterol (mmol/L)	4.1 ± 1.3	4.4 ± 1.1	0.647	0.522
LDL (mmol/L)	2.8 ± 1.1	2.8 ± 0.9	0.225	0.823
HDL (mmol/L)	1.0 ± 0.2	1.0 ± 0.2	0.034	0.978
Triglycerides (mmol/L)	2.1 ± 2.5	1.5 ± 0.8	–0.857	0.398
Homocysteine (μmol/L)(mmol/L)	15.1 ± 8.2	13.5 ± 5.2	–0.674	0.505
Phospholipase-A2 (ng/ml)	299.4 ± 94.9	278.6 ± 105.1	–0.592	0.558
WBC (/L)	6.5 ± 1.0	6.6 ± 1.5	0.298	0.767
Time (*h*)	54.5 ± 18.8	59.5 ± 23.5	–0.669	0.509
Right stenotic MCA (%)	9 (60%)	13 (72%)		0.488^a^

^a^*The *P*-values are obtained by using Fisher exact test. HDL, high-density lipoprotein; LDL, low-density lipoprotein; WBC, white blood cell count; Time, time from admission to HR-MRI examination. Data are presented as number (%) or mean ± SD*.

In total 33 patients, PR was found in 14 patients, the other 19 patients had NR or non-remodeling, at the stenotic MCA site. The differences of sex, age, smoker, alcoholism, hypertension, diabetes, blood glucose, HbA1c, total cholesterol, LDL, HDL, triglycerides, homocysteine, phospholipase-A2, white blood cell count, time from admission to HR-MRI examination, location of stenosis between PR group and non-PR group also exhibited no statistical significances (**Table 2**).

**Table 2:** Demographic and clinical characteristics of PR group and non-PR group.

	PR group (*n* = 14)	Non-PR group (*n* = 19)	*t*-value	*P*-value
Male (%)	12 (86%)	15 (79%)		1.000^a^
Age (years)	66 ± 12	67 ± 12	–0.847	0.403
Smoker (%)	7 (50%) 90	10 (53%)		1.000^a^
Alcoholism (%)	7 (50%)	8 (42%)		0.733^a^
Hypertension (%)	10 (71%)	11 (58%)		0.486^a^
Diabetes (%)	9 (64%)	10 (53%)		0.723^a^
Blood glucose (mmol/L)	5.9 ± 1.6	6.0 ± 1.7	–0.152	0.880
HbA1c (%)	6.9 ± 1.5	6.2 ± 1.3	1.091	0.284
Total cholesterol (mmol/L)	4.2 ± 1.2	4.3 ± 1.1	–0.230	0.820
LDL (mmol/L)	2.8 ± 1.1	2.9 ± 0.9	–0.401	0.691
HDL (mmol/L)	0.9 ± 0.2	1.2 ± 1.0	–1.082	0.288
Triglycerides (mmol/L)	2.4 ± 2.6	1.6 ± 1.3	1.105	0.278
Homocysteine(μmol/L)	14.5 ± 8.2	13.9 ± 5.7	0.221	0.826
Phospholipase-A2 (ng/ml)	276.7 ± 107.4	287.1 ± 113.9	–0.266	0.792
WBC (/L)	6.7 ± 1.1	6.6 ± 1.5	0.352	0.727
Time (h)	56.1 ± 14.6	58.1 ± 25.6	–0.260	0.797
Right stenotic MCA (%)	11 (79%)	12 (63%)		0.455^a^

^a^*The *P*-values are obtained by using Fisher exact test. HDL, high-density lipoprotein; LDL, low-density lipoprotein; WBC, white blood cell count; Time, time from admission to examination. Data are presented as number (%) or mean ± SD*.

### Quantitative Measurement and Calculation of the MCA

**FIGURE 1:**
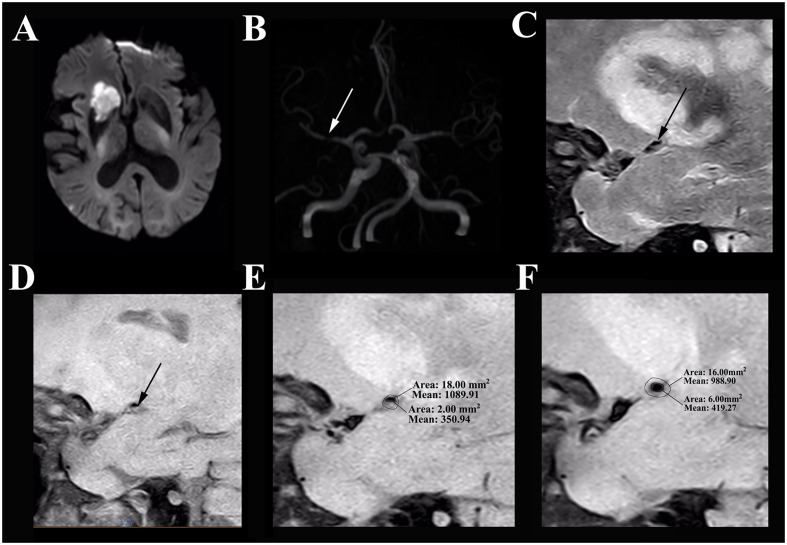
**HR-MRI of a symptomatic MCA stenosis in an 85-year-old male who presented with left limb weakness for 10 hours. (A)** DWI shows an acute ischemic stroke in the distribution of the right MCA. **(B)** The TOF-MRA manifests severe stenosis of the M1 segment of right MCA (white arrow). **(C)** The plaque in T2-weighted HR-MRI (black arrow). **(D)** The plaque in T1-weighted HR-MRI (black arrow). **(E)**. Measurement at the most narrowed site in proton density-weighted HR-MRI: the vessel area is 18.00 mm^2^ and the lumen area is 2.00 mm^2^. **(F)** Measurement at the reference site in proton density-weighted HR-MRI: the vessel area is 16.00 mm^2^ and the lumen area is 6.00 mm^2^. The remodeling index = 18.00 mm^2^/16.00 mm^2^= 1.13 (positive remodeling); the degree of stenosis = (1–2.00 mm^2^/6.00 mm^2^) = 66.67%.

**FIGURE 2:**
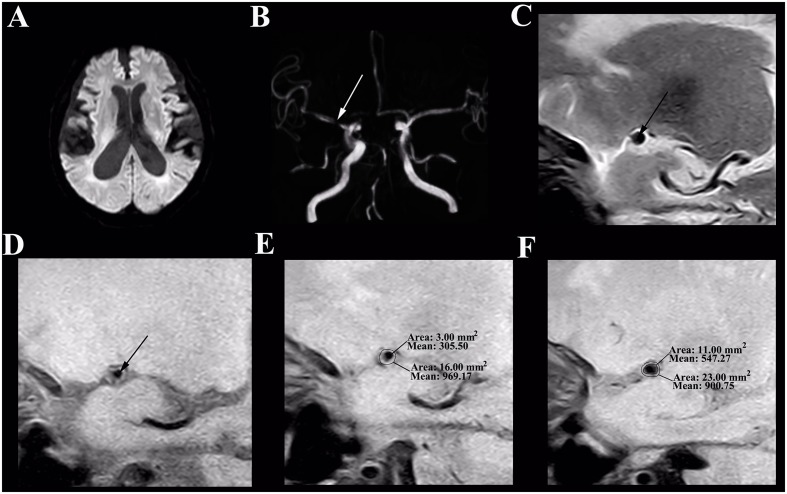
**HR-MRI of an asymptomatic MCA stenosis in an 82-year-old male who presented with aphasia for 1 day. (A)** DWI shows normal. **(B)** The TOF-MRA manifests severe stenosis of the M1 segment of right MCA (white arrow). **(C)** The plaque in T2-weighted HR-MRI (black arrow). **(D)** The plaque in T1-weighted HR-MRI (black arrow). **(E)** Measurement at the most narrowed site in proton density-weighted HR-MRI: the vessel area is 16.00 mm^2^ and the lumen area is 3.00 mm^2^. **(F)** Measurement at the reference site in proton density-weighted HR-MRI: the vessel area is 23.00 mm^2^ and the lumen area is 11.00 mm^2^. The remodeling index = 16.00 mm^2^/23.00 mm^2^= 0.70 (negative remodeling); the degree of stenosis = (1–3.00 mm^2^/11.00 mm^2^) = 72.73%.

The differences of MCA stenosis between the symptomatic group and the asymptomatic group were illustrated in **Table 3**. Moreover, the wall characteristics and remodeling patterns of each group from the HR-MRI were shown in **Figures 1**, **2**. The RI and the degree of MCA stenosis in the symptomatic group were higher than that in the asymptomatic group (*P* < 0.01; *P* = 0.038). At the most narrowed lumen, the WA and PA of the symptomatic group were larger than that of the asymptomatic group (*P* = 0.04; *P* < 0.01). The LA of the symptomatic group was smaller than that of the asymptomatic group (*P* < 0.01). The WA, LA, VA at the reference site, and the VA of the most narrowed lumen did not have any significant differences between groups. The NIHSS score in the symptomatic group was larger than that in the asymptomatic group (*P* = 0.003). In particular, the PR in the symptomatic group was higher than that in the asymptomatic group (*P* = 0.002).

**Table 3:** Quantitative data of the MCA wall in symptomatic group and asymptomatic group.

	Symptomatic group (*n* = 15)	Asymptomatic group (*n* = 18)	*t*-value	*P*-value
Vessel area _reference_ (mm^2^)	15.34 ± 3.11	16.22 ± 3.33	0.776	0.443
Lumen area _reference_ (mm^2^)	6.26 ± 1.38	6.76 ± 1.71	0.901	0.374
Vessel area _MNL_ (mm^2^)	16.22 ± 2.85	15.04 ± 2.60	–1.242	0.223
Lumen area _MNL_ (mm^2^)	2.38 ± 0.71	3.25 ± 0.69	3.543	0.001^∗^
Wall area _reference_ (mm^2^)	9.08 ± 2.02	9.46 ± 2.04	0.538	0.594
Wall area _MNL_ (mm^2^)	13.84 ± 3.05	11.79 ± 2.44	–2.141	0.040^∗^
Plaque area_MNL_ (mm^2^)	4.76 ± 2.00	2.33 ± 1.32	–4.178	0.000^∗^
Degree of stenosis (%)	60.40 ± 12.24	50.61 ± 13.44	–2.169	0.038^∗^
Remodeling index	1.07 ± 0.09	0.94 ± 0.08	–4.481	0.000^∗^
NIHSS	4.87 ± 2.88	1.44 ± 3.07	3.280	0.003^∗^

∗*P-value < 0.05. MNL, most narrowed lumen; Reference, reference site; NIHSS, National Institute of Health stroke scale. Data are presented as mean ± SD*.

The differences of MCA stenosis between the PR group and the non-PR group were illustrated in **Table 4**. The PA of the PR group was larger than that of the non-PR group (*P* = 0.014); the NIHSS score was higher in the PR group (*P* = 0.037). There were 12 symptomatic patients in the PR group, three symptomatic patients in the non-PR group, the difference had statistical significance (*P* < 0.01).

**Table 4:** Quantitative data of the MCA wall in PR group and non-PR group.

	PR group (*n* = 14)	Non-PR group (*n* = 19)	*t*-value	*P*-value
Vessel area_referrence_ (mm^2^)	14.72 ± 3.16	16.63 ± 3.08	-1.741	0.092
Lumen area _referrence_ (mm^2^)	6.09 ± 1.30	6.86 ± 1.70	-1.422	0.165
Vessel area _MNL_ (mm^2^)	15.65 ± 3.03	15.52 ± 2.59	0.135	0.894
Lumen area_MNL_ (mm^2^)	2.60 ± 0.76	3.05 ± 0.82	-1.611	0.117
Wall area _referrence_ (mm^2^)	8.63 ± 2.12	9.77 ± 1.83	-1.651	0.109
Wall area _MNL_ (mm^2^)	13.06 ± 3.28	12.47 ± 2.62	0.569	0.574
Plaque area_MNL_ (mm^2^)	4.42 ± 2.05	2.70 ± 1.75	2.594	0.014^∗^
Degree of stenosis (%)	56.00 ± 13.51	54.37 ± 14.05	0.335	0.740
NIHSS	4.43 ± 3.37	1.95 ± 3.12	2.184	0.037^∗^

∗*P-value < 0.05. MNL, most narrowed lumen; Reference, reference site; NIHSS, National Institute of Health stroke scale. Data are presented as mean ± SD*.

## Discussion

The current study demonstrated that the symptomatic group had larger wall area, plaque area, degree of stenosis, RI, NIHSS score, and smaller lumen area than the asymptomatic group while more PR patients were found in the symptomatic group. Especially, the PR group had larger plaque area and NIHSS score than the non-PR group, which identified that the PR was an unsafe remodeling way and was prone to cause acute ischemic stroke. We suggested that the plaque burden and remodeling way might have direct correlations with the clinical symptom in patients with atherosclerotic MCA stenosis. Meanwhile, this study proved that HR-MRI can be used to depict the vessel wall of stenotic MCA clearly and accurately without any damage to patients, which is therefore considered as a promising tool to detect the characteristics of intracranial arteries wall.

The arteries are dynamic organs, which could change their morphology for compensation when atherosclerotic plaques forming (Fukuda et al., 2014). This arterial adaptation to plaque development was first found by Glagov et al. (1987) in earlier studies of coronary arteries, which was known as Glagov phenomenon or arterial remodeling, including PR and NR. The PR resulted in the vessel enlargement and the NR restricted the vessel. Meanwhile, the PR was strongly associated with symptomatic patients while the NR was more common in asymptomatic patients in both coronary and basilar arteries (Miao et al., 2009; Ma et al., 2010). The MCA wall contains the same intima, media, adventitia as coronary arteries and carotid arteries, although the relative thickness of each component is different (Shi et al., 2012; Zhao et al., 2015). Anyway, the MCA atherosclerotic stenosis might have similar vascular biological features with the aforementioned arteries.

With the development of MR scanner and black blood technique, the HR-MRI has been used to depict the wall characteristics of MCA (Sui et al., 2015). Intracranial atherosclerotic stenosis was the most common reason of stroke in Asia (Wong and Li, 2003; Perren et al., 2015), especially in China (Wang et al., 2014), thus, most studies were conducted in Asian countries. Previous studies have found that contrast enhancement of atherosclerotic MCA plaques might serve as a marker of plaques’ stability using HR-MRI (Qiao et al., 2014; Teng et al., 2016). Other researches demonstrated that symptomatic patients had larger vessel area, wall area, plaque burden, and remodeling ratio in stenotic MCA than that in asymptomatic patients, and a higher prevalence of positive PR was found in symptomatic patients (Xu et al., 2010; Chung et al., 2012; Zhu et al., 2013; Zhao et al., 2015, 2016). Our findings were consistent with these studies. However, the concept of the symptomatic group was different in our study. We defined the symptomatic group as hyperintense signal on DWI in distribution of stenotic MCA, which demonstrated that the acute ischemic stroke was associated with higher RI and PR more directly. It suggested that patients with higher RI and PR were in high risk of acute ischemic stroke, they should take intervention therapy positively. Furthermore, the degree of stenosis in the symptomatic group was higher than that of the asymptomatic group in the current study that was in consistent with the study of Chung et al. (2012). However, Zhao et al. (2015) identified that there was no difference in the degree of stenosis between the symptomatic group and the asymptomatic group. We speculated that it might be associated with the differences in group assignment. The symptomatic group had brain parenchyma injury and was accompanied with more severe clinical symptom and MRI findings than the asymptomatic group. In this way, the degree of stenosis might be higher in the symptomatic group.

To the best of our knowledge, few studies have demonstrated the differences of stenotic MCA wall between the PR group and the non-PR group while most usually focused on the symptomatic group and the asymptomatic group. Shi et al. found the PR group had a greater VA and WA at the site of maximal luminal narrowing, and the microembolics were observed more frequently in the PR group than in the non-PR group (Shi et al., 2012). In our study, we also divided patients into the PR group and the non-PR group and obtained the consistent results with Shi et al. (2012). Additionally, we discovered that there was more hyperintense signal on DWI in the PR group than the non-PR group, which could directly illustrate that the PR was an unsafe remodeling way in a sense for patients with atherosclerotic MCA stenosis.

The NIHSS score assessment was developed in order to standardize the severity of stroke for clinical treatment (Lyden et al., 1994). It’s a 15-item examination that takes a very short time by trained personnel and one of the most favored scoring tools used in centers that treat patients who suffer from ischemic stroke (Martin-Schild et al., 2015). The higher NIHSS score suggested a more serious stroke, which leaded to a poorer outcome. Cao et al. (2017) suggested that plaque burden in symptomatic MCA stenosis and extracranial carotid artery stenosis were significantly associated with NIHSS scores, and stronger statistical correlations between NIHSS score and plaque burden were observed in the MCA compared to the ECA. The present study further investigates that the NIHSS was higher in the PR group than in the non-PR group in patients with atherosclerotic MCA stenosis. Therefore, our results indicated that the PR might represent a more biologically active lesion associated with plaque vulnerability and was more likely to lead to cerebral infarction.

Several limitations should be taken into account in interpreting our results. Firstly, the current study is cross-sectional with a relatively small sample size. Therefore, further studies with a larger sample size will be beneficial to establish the relationship between the wall characteristics of stenotic MCA and acute ischemic stroke. Secondly, all the population of our study had moderate or severe MCA stenosis. Nevertheless, we did not include the patients with mild stenotic MCA (< 30%), which implied that our findings were not applicable to all patients with MCA stenosis. Thirdly, the RI depended on the reference vessel area. We chose proximal or distal segments as a reference site, which might cause underestimation or overestimation of the RI due to the natural tapering of the MCA. However, using the average of distal and proximal to the stenosis for remodeling calculation might result in measurement error mainly due to many times of measurement. Accordingly, we should further develop more accurate tools for MCA measurement. Moreover, we did not perform a follow-up study to investigate whether the treatment effect of PR group was better than non-PR group. The results of clinical treatment on symptomatic and asymptomatic patients will be collected in further study. Finally, although patients with ipsilateral carotid stenosis more than 50% were excluded, we also could not completely exclude the influence from carotid plaques with the stenosis of less than 50%. The ultrasound can be used to monitor carotid plaques and minimize the impact of carotid plaques. This confounding factor should be taken into consideration in our future studies.

## Conclusion

In summary, several features of stenotic MCA wall, including larger WA, PA, RI, and PR were observed more frequently among symptomatic patients with acute ischemic stroke in distribution of stenotic MCA than asymptomatic patients. As an unsafe remodeling pattern, patients with PR at the most narrow-site of MCA had larger PA than non-PR group, especially reflecting more severe neurological deficits. The current study proves the HR-MRI as a promising tool to detect the characteristics of intracranial arteries wall and reveal the relationship between remodeling patterns and ischemic stroke.

## Author Contributions

D-FZ and Y-CC designed the experiment, collected the data, performed the analysis, and wrote the paper. HC, C-NM, WS, and PW helped collect the data and perform the analysis. W-DZ, JS, and XY contributed to the discussion and manuscript revision.

## Conflict of Interest Statement

The authors declare that the research was conducted in the absence of any commercial or financial relationships that could be construed as a potential conflict of interest.
